# Pyoderma Gangrenosum Mimicking an Infected Wound following Dynamic Hip Screw Fixation

**DOI:** 10.1155/2015/571472

**Published:** 2015-08-24

**Authors:** Metin Nizamoglu

**Affiliations:** Trauma & Orthopaedics Department, Leighton Hospital, Middlewich Road, Crewe, Cheshire CW1 4QJ, UK

## Abstract

Pyoderma gangrenosum (PG) is an inflammatory ulcerative neutrophilic dermatosis that can occur following skin trauma. The correct diagnosis is not often made immediately as the condition can mimic an infective appearance. This leads to delays in the appropriate management of high dose steroids. Although debridement can offer aid in resolving lesions, this is contraindicated in the acute phase as this can cause acceleration of the pathogenic process. Biopsy of the lesion does not offer a definitive diagnosis; therefore suspicion must be maintained as the diagnosis is ultimately a clinical one. Any postoperative pustular ulcerative lesion not improving despite antibiotic therapy that also yields negative bacteriological and fungal studies should lead to consideration of this diagnosis. We document the first case of PG developing following intertrochanteric femur fracture fixation using dynamic hip screw.

## 1. Introduction

Pyoderma gangrenosum (PG) is a rare skin disease. It has been noted that PG can arise spontaneously or following skin trauma, including surgery. It has been described as a complication following many different surgical procedures, such as breast surgery [1], cardiothoracic surgery [2], and surgery of the limbs and extremities [3]. Although pyoderma gangrenosum has been documented following hip surgery [4], our literature search has not identified a case following dynamic hip screw fixation for intertrochanteric femur fracture. PG is an aseptic inflammatory ulcerative neutrophilic dermatosis of unknown aetiology [5]. Clinically it starts with sterile pustules that rapidly progress into painful ulcers of variable depth and size, with undermined violaceous borders. It is also associated with a mucopurulent or haemorrhagic exudate [6]. Following surgery diagnosis and subsequent management of PG are often delayed as it often mimics a wound infection. The management of PG is not with antibiotics or wound debridement (the latter being contraindicated acutely); rather it is with high dose steroids and immunosuppressive agents. Therefore it is essential to be aware of this complication in order to recognise it and begin the appropriate treatment, preventing unnecessary wound debridement that can accelerate the pathological process. We describe a case of PG developing following dynamic hip screw fixation in a patient with an intertrochanteric femur fracture.

## 2. Case

**Figure 1:**
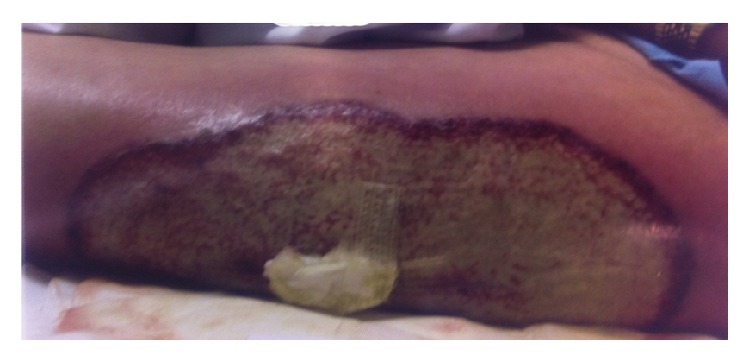
PG at DHS wound site.

We present the case of PG secondary to dynamic hip screw fixation for traumatic intertrochanteric femur fracture in an 82-year-old male following a mechanical fall at home. His past medical history consisted of hypertension, benign prostatic hypertrophy, myocardial infarction 16 years prior to admission, atrial fibrillation, recurrent episodes of supraventricular tachycardia, and a cardiac defibrillator in situ. The day following admission the fracture was reduced on traction table and dynamic hip screw fixation was performed under a spinal anaesthetic. The cardiac defibrillator was switched off during the procedure and the operation was deemed satisfactory. There were no intraoperative complications reported. The wound was closed using surgical staples. Postoperatively the patient's haemoglobin was 7.1 g/L. He was transfused 2 units of packed red cells. A catheter was inserted on the 2nd day of surgery as the patient went into acute urinary retention. On the 8th day following surgery the patient developed a drop in saturation to 93%, with a temperature of 38 degrees Celsius. White blood cell count (WCC) was 18.1. Neutrophil count was 16.17. C-reactive protein (CRP) was 165. His chest X-ray findings were consistent with consolidation. He was started on intravenous piperacillin/tazobactam antibiotics for hospital acquired pneumonia. On the 10th day of surgery the patient's temperature spiked to 39 degrees Celsius. On inspection the wound appeared erythematous, hot, tender, tense, and fluctuant on palpation, with a purulent discharge between surgical staples. His WCC had risen to 37.11 and his CRP had risen to 227. Intravenous antibiotics were changed to teicoplanin as per microbiologist advice. The distal 2 staples of the wound were removed to facilitate drainage of pus. Blood cultures and wound swabs were taken. At this point consideration began for debridement and washout of the wound. The wound swabs and blood cultures showed no organism growth. On the 17th day of surgery it was noted the skin was breaking down around the wound. This began with 3 small pustules located around the incision site, and these enlarged and developed into circular ulcers. An ultrasound scan confirmed a collection beneath the wound. On the 22nd day of surgery the patient was taken to theatre for washout and debridement of the wound as no improvement was seen with conservative measures. Despite the wound being washed out in theatre and intravenous antibiotics the ulcerated lesions continued to enlarge. The 3 circles noted earlier then merged into a single large ulcerated lesion with a pale central area and a clear demarcated dark violaceous border (Figure 1). The patient was seen by the dermatologist and tissue biopsies were taken. The diagnosis of PG was made from clinical appearance and prednisolone 1 mg/kg treatment was started immediately, with instructions to be reviewed following 2 days of therapy. Investigations were also conducted for associated pathology such as rheumatoid arthritis, inflammatory bowel disease, and other autoimmune conditions. On the 25th day of surgery the patient's temperature was settling. Blood and wound cultures continued to return negative results, so antibiotics were stopped. The ulcerated lesion then began to regress. The patient began to improve clinically and started mobilizing with the aid of crutches. However on the 30th day of surgery the patient rapidly deteriorated with an increased respiratory rate, reduced saturations and respiratory failure. He was in fast atrial fibrillation and developed pulmonary oedema. The patient was transferred to the intensive care unit where he was restarted on intravenous teicoplanin. The patient continued to deteriorate ultimately leading to death. A postmortem examination was performed. Bronchopneumonia was identified as the primary cause of death, with congestive cardiac failure and ischaemic heart disease as contributing factors.

## 3. Discussion

PG has been well documented as a sequela to surgery. The diagnosis is often delayed as the clinical findings often mimic a wound infection, particularly due to the purulent looking discharge associated with the skin lesions. It is important that PG is diagnosed promptly to prevent further progression of the disease. Six disease categories may imitate the clinical appearance of PG [7]. These are vascular occlusive or venous disease, vasculitis, cancer, infection, drug reaction, and exogenous tissue injury. It is important that the rapid onset of postsurgical PG must be differentiated from acute deep skin infection such as erysipelas or gangrene [8]. Early dermatology review is advised. PG is a clinical diagnosis as there are no sensitive diagnostic tests available. Biopsies of the lesions are nondiagnostic and demonstrate nonspecific areas of necrosis and ulceration, characterized by the infiltration of acute and chronic inflammatory cells [9]. However tissue biopsy is important to exclude infectious diseases that mimic PG even though this may exacerbate the pathogenic process. Biopsied tissue samples should be sent for bacterial and fungal cultures as well as staining for alternate microorganisms as per dermatologist advice. In the instance of misdiagnosed wound infection washout and debridement can facilitate the inflammatory reaction and exacerbate the problem further [10]. Therefore, any surgical procedure has to be done as an adjunct to immunosuppression only in patients with stable disease or partial remission. Autologous split-skin grafts have been used with variable outcome [11].

Corticosteroids are widely used for initial therapy [12]. Cyclosporine A has become an accepted treatment for widespread PG after initial steroids or in combination with steroids. However combination with various other immunosuppressive agents may become necessary if remission is not satisfactory. For sloughy or purulent covered lesions wet compresses with sterile saline solution or Ringer-lactate solution and alginate dressings are useful. Pain relief and improvement of odour have also been observed with compression [12]. Topical corticosteroids can also be applied. Finally, for individuals with a history of PG, prophylactic systemic steroid treatment before and after elective surgery has been recommended [13].

## 4. Conclusion

In summary, the diagnosis of pyoderma gangrenosum should be considered in any postoperative skin lesion. The warning signs are rapidly spreading ulceration of the operative site which does not improve with antibiotic therapy particularly when wound swabs or other bacteriological findings yield negative results. A past medical history of inflammatory bowel disease, arthropathy, previous or family history of PG, and immunological or neoplastic pathology may predispose patients to this condition. Histopathology offers little in the way of diagnosis; therefore this is often established on a clinical level. A bluish/violaceous tinged outline of the lesions should raise suspicion of the diagnosis of PG. It is important to establish the diagnosis early for timely administration of corticosteroids to prevent a severe inflammatory syndrome and induce remission of the disease process. Early dermatology review is advised in cases of suspected PG. It is important for surgeons to recognise and manage this postoperative complication.
